# Development and validation of a novel endoplasmic reticulum stress-related lncRNA prognostic signature and candidate drugs in breast cancer

**DOI:** 10.3389/fgene.2022.949314

**Published:** 2022-08-25

**Authors:** Jiehui Cai, Zeqi Ji, Jinyao Wu, Lingzhi Chen, Daitian Zheng, Yaokun Chen, Xinkang Zhang, Wanchun Xie, Jieying Huang, Manqi Chen, Ru Lin, Weixun Lin, Yexi Chen, Zhiyang Li

**Affiliations:** Department of Thyroid, Breast and Hernia Surgery, The Second Affiliated Hospital of Shantou University Medical College, Shantou, China

**Keywords:** breast cancer, endoplasmic reticulum stress, long non-coding RNA, tumor immune microenvironment, prognostic signature, candidate drugs

## Abstract

Breast cancer (BC), the most common malignancy in women, has a high cancer-related mortality. Endoplasmic reticulum stress (ERS), a response to the accumulation of unfolded proteins, has emerging roles in tumorigenesis, including invasion, metastasis, immune escape, etc. However, few studies have focused on the correlation between ERS with long non-coding RNAs (lncRNAs) in BC. We attempted to construct an ERS-related lncRNA prognostic signature and study its value in BC from tumor mutational burden (TMB), tumor immune microenvironment (TIME), cluster, clinical treatment, and so on. In the present study, transcriptomic and clinical data of BC patients were extracted from The Cancer Genome Atlas (TCGA) database. Correlation test, Cox regression analysis, least absolute shrinkage, and selection operator (LASSO) method were performed to determine an ERS-related lncRNA prognostic signature. Survival and predictive performance were analyzed according to Kaplan–Meier curves and receiver operating characteristic (ROC) curves, while nomograms and calibration curves were established. Then, an enrichment analysis was performed to study the functions and biological processes of ERS-related lncRNAs. TMB and TIME were also analyzed to assess the mutational status and immune status. Additionally, by using consensus cluster analysis, we compared differences among tumor subtypes. Drug sensitivity analysis and immunologic efficacy evaluations were performed together for further exploration. We identified a novel prognostic signature consisting of 9 ERS-related lncRNAs. High-risk patients had worse prognoses. The signature had a good predictive performance as an independent prognostic indicator and was significantly associated with clinicopathological characteristics. Enrichment analysis showed that metabolic pathways were enriched in high-risk patients, while immune pathways were more active in low-risk patients. Low-risk patients had lower TMB, higher immune scores, and stronger immune functions. Cluster analysis clarified that cluster 2 had the most active immune functions and was sensitive to more drugs, which may have the best clinical immunological efficacy. A clinical efficacy evaluation revealed that patients in the low-risk group may benefit more from chemotherapy, targeted therapy, and immunotherapy. The novel signature has significant clinical implications in prognosis prediction for BC. Our study clarifies that there is a potential connection between the ERS-related lncRNAs and BC, which may provide new treatment guidelines for BC.

## Introduction

Breast cancer (BC) is the most prevalent malignant tumor among females and the leading cause of female mortality worldwide (Erratum, 2020). With the new molecular hallmarks being extensively explored, significant progress for BC drug therapy has been made in recent years, including targeted therapies such as CDK4/6 (cyclindependent kinase 4/6) inhibitors, anti-programmed cell death ligand 1 (PD-L1) immunotherapy, phosphatidylinositol 3-kinase inhibitors (PI3K), chemotherapy, and endocrine therapy (Loibl et al., 2021). However, there is a big challenge in BC diagnosis and treatment because of its high recurrence and metastasis rate (Liu et al., 2017). The five-year survival rate for BC is about 85%, and the prognosis for advanced BC is even worse (Mei et al., 2020). Therefore, an in-depth understanding of the mechanisms underlying the occurrence and progression of BC is essential for developing more effective treatments and improving clinical outcomes.

Endoplasmic reticulum stress (ERS) is the response to the accumulation of unfolded proteins and an imbalanced Ca2+ concentration, which results in an ER homeostasis imbalance (Yao et al., 2020a; Li et al., 2021). It is reported that ERS, as a defense system, can be induced by multiple physiological and pathological conditions such as hypoxia, oxidative stress, chronic inflammation, and imbalance in calcium homeostasis (Hotamisligil, 2010; Song et al., 2019); whereas, excessive or persistent ERS will lead to autophagy and apoptosis (Lee et al., 2020; Song et al., 2020). What’s more, ERS can activate a series of signal transductions and regulations to restore the ER protein balance, which is an adaptive response called the unfolded protein response (UPR) (Hetz, 2012). The emerging role of UPR has been observed in tumorigenesis, including angiogenesis, tumor growth, invasion, metastasis, immune escape, and resistance to chemotherapy and radiotherapy (Urra et al., 2016). The cell defense mechanisms induced by stress are essential for mediating tumor resistance. ERS has been associated with drug resistance in several malignancies, including multiple myeloma (Nikesitch et al., 2018) and BC (Zhong et al., 2017). Recent research has shown that 5-fluorouracil (5-FU) upregulated the expression of drug resistance-related regulatory factors in BC by inducing ERS activation, thus enhancing cell resistance to 5-FU (Yao et al., 2020b). Above all, it is indicated that ERS may be a potential therapeutic target for malignant tumor treatment.

Long non-coding RNA (LncRNA), with a length of ≥200 nucleotides, is a type of RNA molecule transcribed from the genome and takes part in the regulation of protein coding genes or other non-coding RNA family members (Rinn and Chang, 2020; Tsagakis et al., 2020; Statello et al., 2021). LncRNAs are abnormally expressed in a wide range of cancers (Huarte, 2015; Peng et al., 2020), which participate in the epithelial mesenchymal transition (EMT), vascular metastasis, tumor colonization, and other processes, playing a key role in regulating tumor metastasis, and may become a new target for tumor treatment (Ming et al., 2021). LncRNAs are closely related to the occurrence and development of BC. For example, Linc-ZNF469-3 was found to induce EMT by regulating the expression of ZeB-1 and promoting lung metastasis in triple-negative BC (Wang et al., 2018). However, the association between BC- and ERS-related lncRNAs is unclear, which is going to be further explored in this study. Furthermore, the function and biological pathway, and the immune microenvironment will be analyzed to explore the underlying mechanism of ERS in BC. Finally, ERS-related lncRNAs in predicting drug sensitivity and clinical immune efficacy will be evaluated. This research aims to clarify the role of ERS-related lncRNAs in BC, which not only provides a theoretical basis for further exploration of ERS molecules and mechanisms, but may also provide novel ideas for the clinical prognosis and treatment of BC.

## Materials and methods

### Data and gene acquisition

The RNA transcriptome dataset made up of 1,222 human breast tissue samples was obtained from The Cancer Genome Atlas (TCGA, https://portal.gdc.cancer.gov/), which includes 1,109 BRCA samples and 113 normal samples. Related clinical information of BC samples and normal samples were also downloaded from TCGA together. We obtained the gene transfer format (GTF) files from Ensembl (http://asia.ensembl.org) (Howe et al., 2021), which were used to distinguish lncRNA and messenger RNAs (mRNA). From the Gene Set Enrichment Analysis (GSEA) (http://www.gsea-msigdb.org/gsea/index.jsp) database, 3 genesets related with ERS were acquired by searching the keywords “IRE1”, “PERK”, and “ATF6”, respectively, as the corresponding systematic names were “M10426”, “M42776”, and “M5987”. A total of 163 ERS-related genes were obtained for the next identification (Supplementary Table S1).

### Identification of ERS-related lncRNAs and construction of the prognostic signature

Gene expression matrices were constructed using the limma R package. The Pearson correlation coefficient |cor|>0.5, *p* < 0.001 were viewed as the standards to identify ERS-related lncRNAs. With the standard of |log2 fold change (FC) | >1 and the false discovery rate (FDR) <0.05, we screened ERS-related differentially expressed lncRNAs for further analysis by running the limma R package. And, the pheatmap R package was used to draw the heatmap and volcano plot for visualization. In the interest of further exploring ERS-related lncRNAs, we first used the univariate Cox regression analysis to screen out lncRNAs that were significantly associated with the prognosis of BC patients (*p* < 0.05). Also, we drew a forest map and heatmap to clearly assess the impact of these screened lncRNAs on the prognosis. Then, the LASSO regression analysis was used to further filter the lncRNAs, which prevented the risk model of overfitting. Finally, we performed the multivariate Cox proportional hazards regression analysis to construct an ERS-related lncRNA prognostic signature, which was combined with the minimum Akaike Information Criterion (AIC) value (Vrieze, 2012). The survival, caret, glmnet, survminer, timeROC, and pheatmap R packages were used for the aforementioned analyses. The risk score of BC patients was calculated based on the following formula:

$$\mathrm{Risk score}=\sum_{k=1}^{\mathrm{16}}\mathrm{Coef}\left(k\right)\times E\left(k\right)$$

Coef(k) and E(k) represent the abbreviations of the prognostic ERS-related lncRNAs regression coefficient and the expression level of lncRNAs, respectively. And meanwhile, the median value of the risk score was used as a cutoff to classify BC patients into high-risk and low-risk groups.

### Internal validation of the ERS-related lncRNA prognostic signature

For the purpose of assessing the prognostic role of the signature, we conducted the Kaplan–Meier log-rank test to compare the overall survival (OS) between different risk groups. With the pheatmap R package, we also plotted risk curves, survival status maps, and risk heatmaps of the complete, training, and validation sets to further analyze the impact of selected ERS-related lncRNAs on prognosis. Additionally, the independent prognostic analysis was performed to identify the prognostic value of the signature. During this process, 183 BC patients whose clinical data were unable to be evaluated were excluded including 24 “stage”, 150 “M" stage, and 9 “N" stage as parts of the clinicopathological characteristics. As a result, 914 BC patients were retained. Next, for evaluating the predictive performance of the ERS-related lncRNA prognostic signature and clinicopathological characteristics in predicting the survival at 1, 3, and 5 years, we drew the receiver operating characteristic (ROC) curve and calculated the area under the curve (AUC) in the way of running the survival, survminer, and timeROC package with the R software. For the sake of further studying the value of the prognostic signature, we plotted Kaplan–Meier survival curves to analyze the correlation between clinicopathological characteristics and the OS in high- and low-risk groups.

### Construction of the nomogram and calibration curves

The nomogram, a predictive tool, is widely used in oncology and medicine (Balachandran et al., 2015). On the basis of the risk score and clinicopathological characteristics including age, gender, stage, and TMN stage, with the regplot, survival, and rms R packages, we set up the nomogram of 1, 3, and 5-year OS. Simultaneously, calibration curves were constructed according to the Hosmer–Lemeshow test to judge the accuracy of the nomogram for clinical prognosis by means of evaluating the fitting degree of the predicted results and the actual observed results.

### Function and biological pathway enrichment analyses

|log2 fold change (FC) > 1| and a false discovery rate (FDR) < 0.05 were used as the criteria to screen out differentially expressed genes in the high- and low-risk groups. By running the “ggplot2” R package, we performed the Gene Ontology (GO) analysis to explore the functions and biological processes of ERS-related lncRNAs, plotted the histogram, and bubble chart as well. Furthermore, so as to identify the biological pathways associated with the ERS-related lncRNAs, we used the GSEA to evaluate differences between patients in the high- and low-risk groups, and regarded *P*< 0.05 and FDR<0.05 as the criteria to analyze these biological pathways, provided by the Kyoto Encyclopedia of Genes and Genomes (KEGG).

### Study of the correlation between TMB and prognostic signature

TMB is the total number of somatic gene coding errors, base substitutions, insertions, or deletions detected across per million bases (Zhang et al., 2019). In view of the Perl script, we calculated the mutation frequency and the number of variants in each sample, and divided all the samples into a high-TMB group and a low-TMB group which were combined with the patients’ survival information analyses. At the same time, we analyzed the difference in survival between the two groups. We also evaluated the mutational status of the genes in the high- and low-risk groups and plotted waterfall plots for visualization. We further conducted a TMB variance analysis and correlation analysis for the high- and low-risk groups by using risk score, limma, ggpubr, ggplot2, and ggExtra R packages. Moreover, in order to exploring the effect of risk score and TMB on the survival of patients, we jointly analyzed the survival differences among the high TMB + high risk group, high TMB + low risk group, low TMB + high risk group, and low TMB + low risk group by means of plotting the Kaplan–Meier curve.

### Evaluation of the tumor immune microenvironment, immune cell infiltration, and function

For the sake of comparing the abundance of cellular infiltrations between the high- and low-risk groups, the estimate and limma R packages were used to calculate the immune cell and stromal cell fractions for each sample. It was the Wilcoxon rank sum test that was used to analyze the differences in StromalScore, ImmuneScore, and ESTIMATEScore between the two groups. Then, so as to further explore the differences in the tumor immune microenvironment between the high-risk and the low-risk groups, we calculated the proportion of infiltrating immune cells in the BC samples on the basis of the CIBERSORT algorithm (Newman et al., 2015). Along with the application of various algorithms which included TIMER (Li et al., 2017), CIBERSORT (Chen et al., 2018), CIBERSORT-ABS (Wang et al., 2020), QUANTISEQ (Plattner et al., 2020), MCPCOUNTER (Shi et al., 2020), XCELL (Aran, 2020), and EPIC (Racle et al., 2017), we also explored the correlation between immune cells and risk scores, and the result was displayed in a bubble chart. Meanwhile, we performed survival analysis of immune cell infiltration using limma, survival, and survminer R packages, screened the results with *P*< 0.05, and drew Kaplan–Meier curves. In addition, we scored infiltrating immune cells and immune-related functions in the BC samples and plotted multi-box plots to analyze the differences between the two groups.

### Cluster analysis of ERS-related lncRNAs

According to the expression levels of 9 ERS-related lncRNAs with predictive value obtained by the multivariate Cox regression analysis and the “ConensusClusterPlus” R package, we performed the consensus cluster analysis on the tumor samples and divided them into different subgroups. The cluster was performed in view of the following criteria. First, the cumulative distribution function curve increased steadily. Second, there were no groups with small sample sizes. Third, the intra-group correlation increased while the inter-group correlation decreased after the cluster. Then, we used the survival and survminer R packages (Zhang et al., 2020) and plotted the Kaplan–Meier curve to analyze the differences in survival of the classified samples. Also, we run dplyr, ggplot2 and ggalluvial to generate the Sankey plot in order to figure out the relationship between sample subgroups and risk. Furthermore, the t-distributed Stochastic Neighbor Embedding (tSNE) analysis was performed on high- and low-risk groups and different subgroups, which can visually observe the grouping of samples. As the characteristics of the immune microenvironment are closely related to the tumors, we compared the StromalScore, ImmuneScore, and ESTIMATEScore between the different subgroups. And using the limma R package, combined with multi-algorithms including TIMER (Li et al., 2017), CIBERSORT (Chen et al., 2018), CIBERSORT-ABS (Wang et al., 2020), QUANTISEQ (Plattner et al., 2020), MCPCOUNTER (Shi et al., 2020), XCELL (Aran, 2020), and EPIC (Racle et al., 2017), we analyzed the immune response differences between different subgroups. With the pheatmap R package, a heatmap was generated for visualization. Furthermore, we analyzed the expression of 43 immune checkpoint inhibitor (ICI)-related immunosuppressive molecules in different subgroups. It was the pRRophetic package that was used to analyze a BC patient’s response to treatment in different subgroups as determined by the half-maximal inhibitory concentration (IC50) on the Genomics of Drug Sensitivity in Cancer (GDSC) (https://www.cancerrxgene.org/), and *p* < 0.001 was set as the criterion to screen out potential drugs.

### The role of ERS-related lncRNAs in predicting drug sensitivity and clinical immune efficacy

For a long time, the research and development of new drugs has been a hot spot in BC treatment study. Based on IC50 of GDSC, we evaluated all BC patients’ responses to treatment as a result of using the pRRophetic R package, screened out potential drugs (*p* < 0.001), and drew boxplots. We applied the ggplot2, ggpubr, limma, and reshape2 R packages to analyze the statistical differences in the aspect of expression levels of common ICI-related immunosuppressive molecules. Apart from it, by the Wilcoxon rank sum test, the differences in immunotherapy efficacy of BC patients between the high- and low-risk groups were assessed, and a boxplot was generated as well.

### Statistical analysis

All analyses in study were conducted using the R software (v.4.1.1). It was *p* value < 0.05 that was considered statistically significant in all the analyses.

## Results

### Screening and identification of ERS-related lncRNAs in BC patients

**FIGURE 1:**
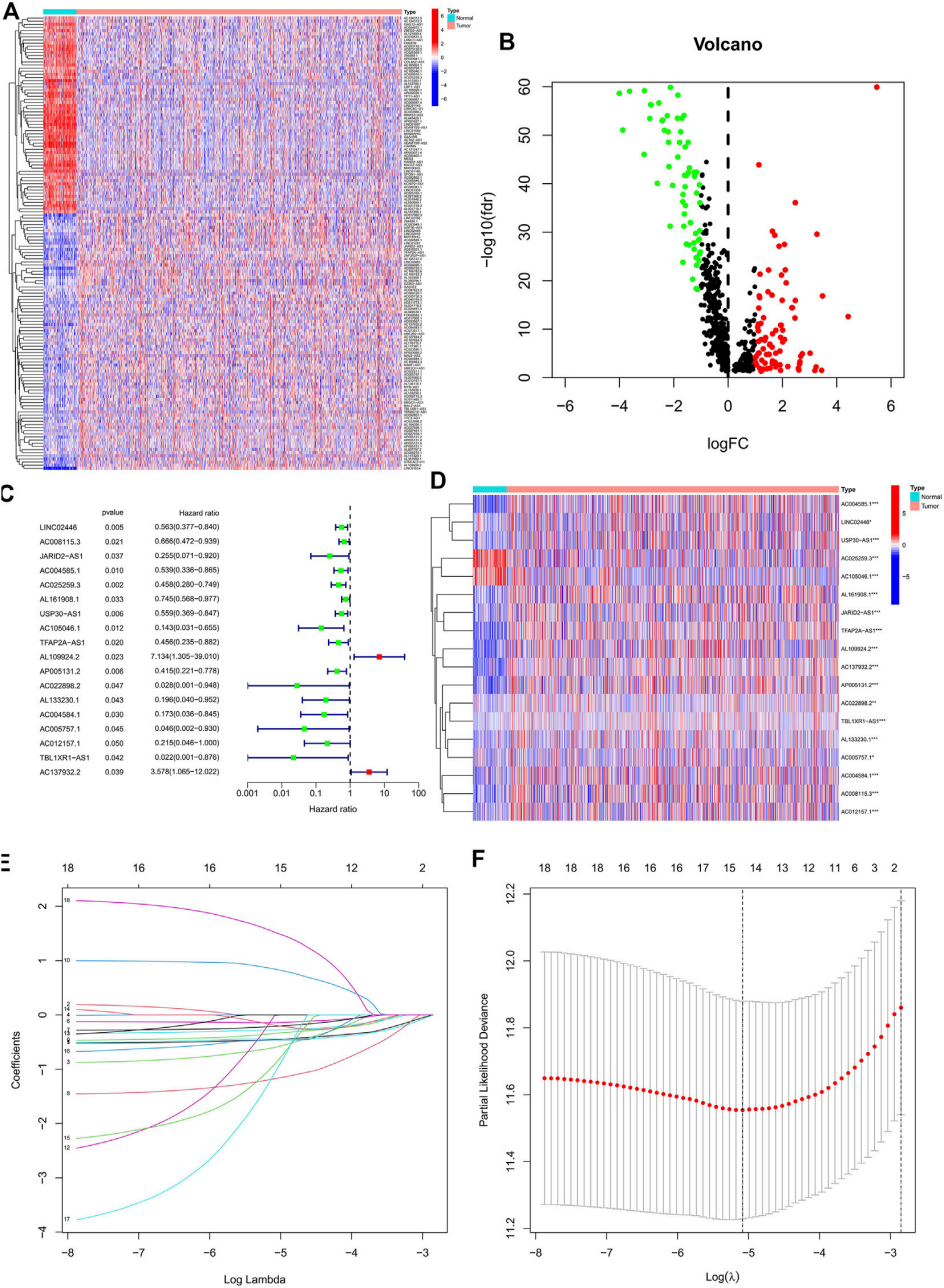
Identification of the prognostic ERS-related lncRNAs for BC patients. **(A)** Heatmap of 145 differentially expressed lncRNAs. **(B)** Volcano plot of ERS-related lncRNAs. Red dots indicate upregulated lncRNAs in tumor tissues while the green dots indicate downregulated lncRNAs. **(C,D)** Forest plot **(C)** and heatmap **(D)** of the prognostic ERS-related lncRNAs extracted by the univariate Cox regression analysis. **(E,F)** LASSO variable trajectory plot for 1,000 cross validations**(E)** and LASSO coefficient profile **(F)**. ERS, endoplasmic reticulum stress; BC, breast cancer.

Based on the TCGA database, we extracted 1,039 cases as those with the survival time missing or less than 30 days were excluded for the follow-up survival analysis. Combining 163 ERS-related genes from public databases, 799 ERS-related lncRNAs (Supplementary Table S2) were finally identified by using the Pearson correlation algorithm. Passing through the differential analysis, we acquired 145 ERS-related lncRNAs (Supplementary Table S3) that were differentially expressed in normal and tumor tissues (|log2 FC| >1 and FDR<0.05). The heatmap showed the expression of 145 ERS-related lncRNAs in normal and tumor tissues (Figure 1A). In the volcano plot of Figure 1B, there were 82 upregulated lncRNAs in the tumor tissue and 63 downregulated lncRNAs.

### Establishment and validation of an ERS-related lncRNA prognostic signature

**FIGURE 2:**
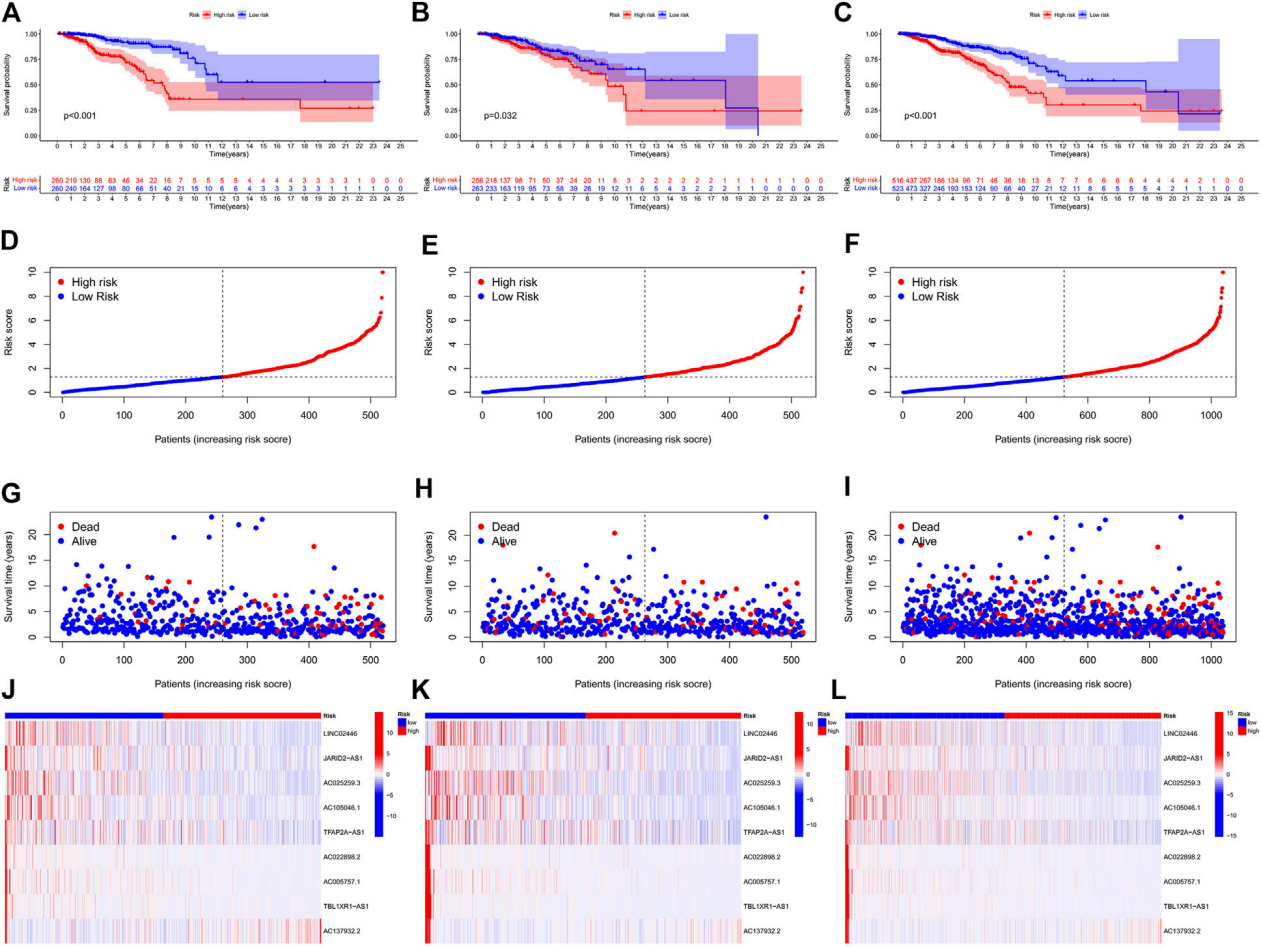
Prognosis and risk scoring analysis of 9 ERS-related lncRNAs in the different sets for BC patients. **(A–C)** Kaplan–Meier survival curves of BC patients’ OS in the high- and low-risk groups in the training **(A)**, validation **(B)**, and complete sets **(C)**. **(D–F)** Risk score distribution in the training **(D)**, validation **(E)**, and complete sets **(F)** for two groups. **(G–I)** Scatter plots of BC patient survival status distribution in the training **(G)**, validation **(H)**, and complete sets **(I)**. **(J–L)** Risk heatmaps of the 9 ERS-related lncRNA expression in the training **(J)**, validation **(K)**, and complete sets **(L)**. Red represents high expression and green represents low expression. ERS, endoplasmic reticulum stress; BC, breast cancer; OS, overall survival.

**FIGURE 3:**
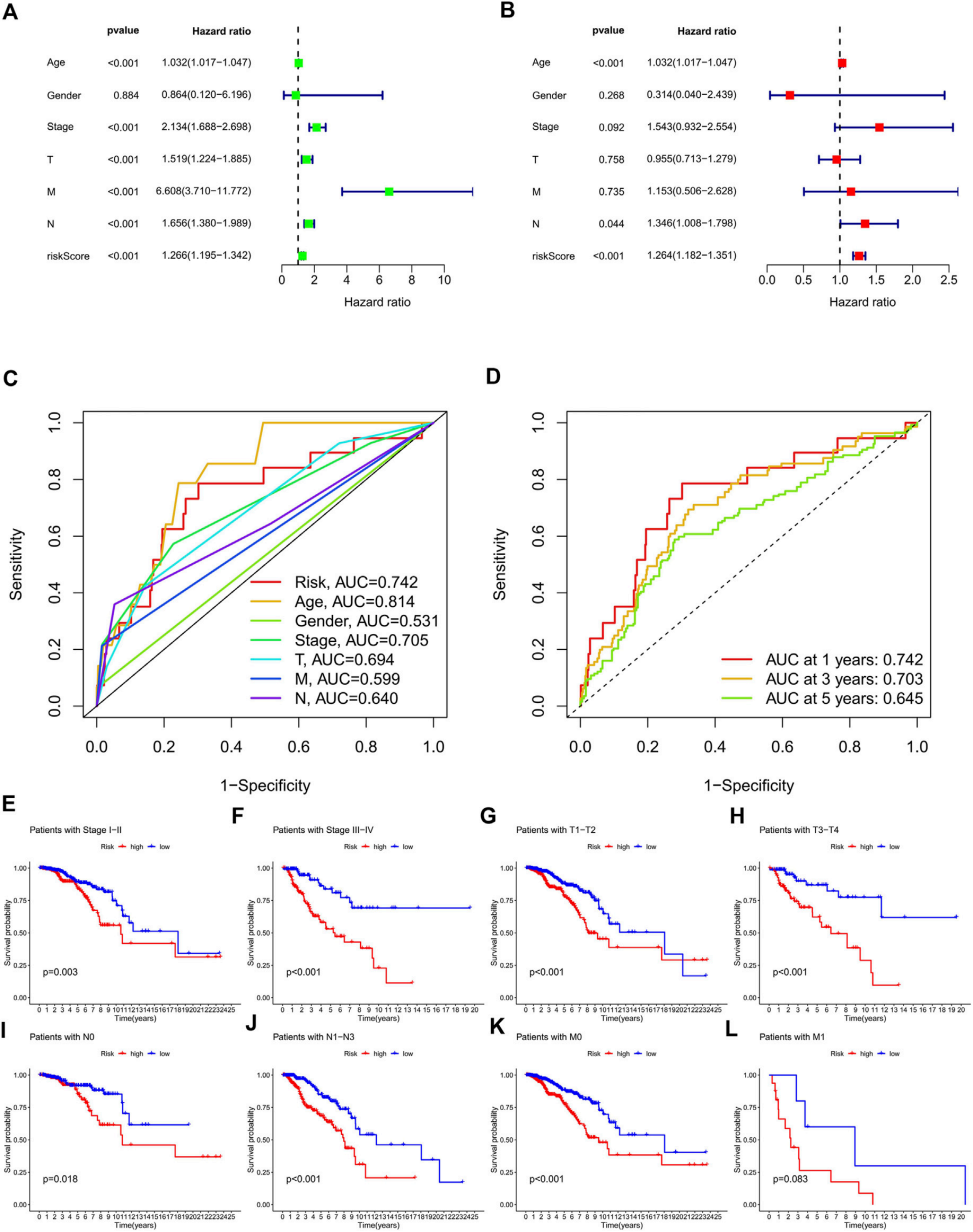
Validation of the ERS-related lncRNA prognostic signature and its relationship with clinicopathological characteristics. **(A,B)** Univariate **(A)** and multivariate **(B)** independent Cox regression analyses of the risk score and clinicopathological characteristics. **(C)** ROC curves and AUCs of the risk score and clinicopathological characteristics. **(D)** ROC curves and AUCs for 1-, 3-, and 5-year survival rates of the complete set. **(E–L)** Kaplan–Meier survival curves of BC patients’ OS on the clinicopathological characteristics between two groups in the complete set. ERS, endoplasmic reticulum stress; AUC, area under the curve; ROC, receiver operating characteristic; OS, overall survival; T, tumor; N, lymph node; M, metastasis.

In the preliminary screening, we extracted 18 ERS-related lncRNAs associated with the BC prognosis using univariate Cox regression analysis. The forest plot (Figure 1C) exhibited the expression difference (*p* value) and Hazard Rates (HR) of 18 ERS-related lncRNAs in BC tissues and normal tissues. From Figure 1D, it can be seen that all other lncRNAs were highly expressed in tumor tissues except AC025259.3 and AC105046.1. Next, we carried out the LASSO regression to penalize 18 ERS-related lncRNAs (Figures 1E,F). Finally, the multivariate Cox regression analysis was performed. As a consequence, the 9 best lncRNAs (LINC02446, JARID2-AS1, AC025259.3, AC105046.1, TFAP2A-AS1, AC022898.2, AC005757.1, TBL1XR1-AS1, and AC137932.2) were identified as prognostic markers for model construction (Table 1). The risk score for each patient was calculated based on the expression level of each lncRNA and the correlation coefficient obtained by the multivariate Cox regression analysis, and taking the median of risk score as the threshold, the patients were separated into high- and low-risk groups. The Kaplan–Meier survival curves showed a statistically significant difference in the overall survival (OS) between the two groups in the training, validation, and complete sets, and the survival rate of the BC patients with high risk was significantly lower than that of BC patients with a low risk (Figures 2A–C), which preliminarily reflected the predictive value of the signature in terms of prognosis for BC patients. The risk score ranking distributions of BC patients are shown in Figures 2D–F in accordance with 9 lncRNAs prognostic markers. It is noteworthy that the scatterplots of the training, validation, and complete sets revealed that the survival status of BC patients was connected with the risk score, and as the risk score increased, the mortality rate of the patients increased (Figures 2G–I). Together with the 9 prognostic ERS-related lncRNAs, we drew heatmaps to compare the expression levels of lncRNAs in the high-risk group and the low-risk group for the training, validation, and complete sets, from which it can be seen that among the different sets, the expression tendency of each lncRNA was basically the same, but the expression level was different in two groups of the same set (Figures 2J–L). With the purpose of confirming whether the prognostic signature can be used as an independent prognostic indicator for BC patients or not, we performed the Cox regression analysis and drew two forest plots on the basis of risk scores and clinicopathological characteristics. The univariate Cox regression analysis showed that almost all factors including age, tumor stage, T stage, N stage, M stage, and model could be used as independent prognostic indicators for BC patients except gender (Figure 3A, *P*< 0.001), while the multivariate analysis showed that age and model were independent prognostic factors of OS in BC patients (Figure 3B, *P*< 0.001). Furthermore, using the ROC curve as a foundation, we observed that the AUC for the risk score was 0.742, whose area was larger compared to other clinicopathological characteristics (gender, stage, T stage, N stage, and M stage) except for age, demonstrating a better predictive performance (Figure 3C). From Figure 3D, we knew that the AUC values for predicting the 1-, 3-, and 5-year survival rates were 0.742, 0.703, and 0.645, respectively, which suggested the potential predictive value of the prognostic signature. As for the correlation between clinicopathological characteristics and the prognostic model, as shown in Figures 3E–L, except for M1 stage (*p* = 0.083), BC patients in the high- and low-risk groups with different clinicopathological characteristics had significant differences in the OS (all *P*< 0.05).

**TABLE 1:** The regression coefficient of 9 ESR-related lncRNAs acquired by the multivariate Cox analysis.

id	Coef
LINC02446	−0.666557169115816
JARID2-AS1	−1.01852389169384
AC025259.3	−0.494972345670693
AC105046.1	−1.70783864216911
TFAP2A-AS1	−0.549326255865056
AC022898.2	−2.95365678990592
AC005757.1	−2.66162430189308
TBL1XR1-AS1	−3.86616790061961
AC137932.2	2.26501183795505

### Construction of the nomogram and biological pathway analyses

**FIGURE 4:**
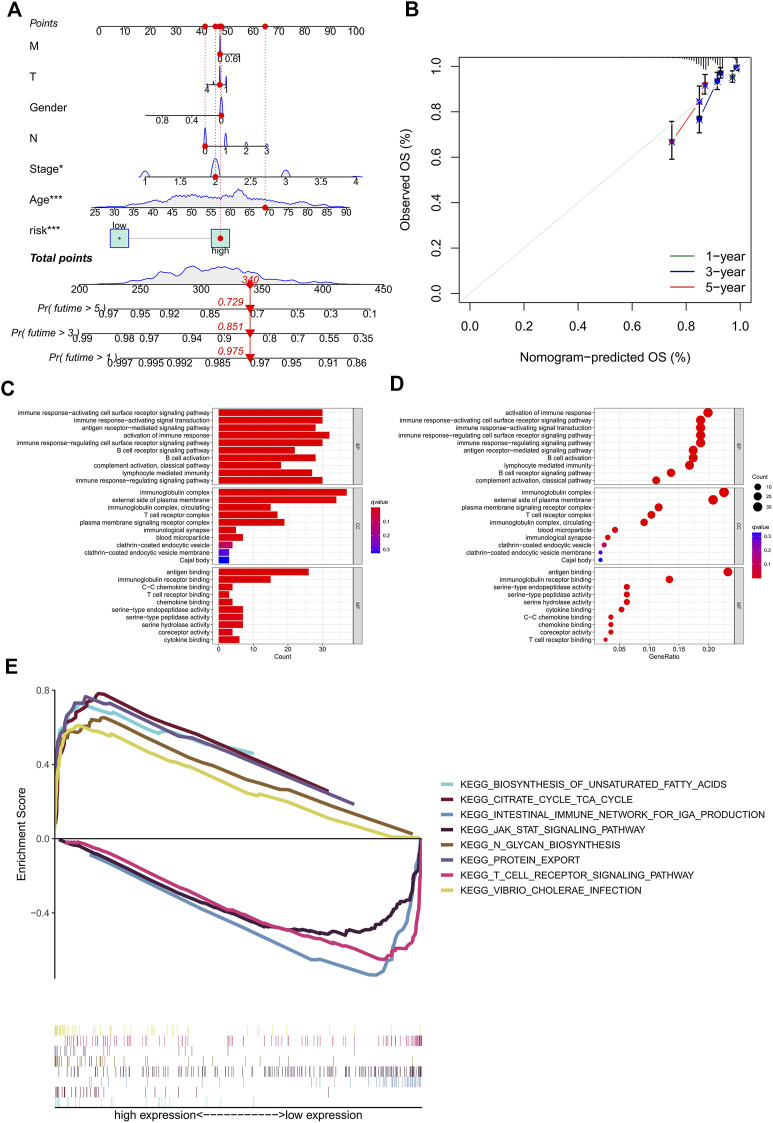
Construction of the nomogram and calibration curves, enrichment analysis of the prognostic signature. **(A)** nomogram prediction model of the combined risk score and clinicopathological characteristics for 1-, 3-, and 5-year OS rate in BC patients. **(B)** Calibration curves for the relationship between predicted survival and the observed OS rate at 1, 3, and 5 years **(C–D)** The GO function enrichment analyses. **(E)** The GSEA pathway enrichment analyses. OS, overall survival; BC, breast cancer.

The nomogram, a quantitative method, was used to predict the 1-, 3-, and 5-year OS of BC patients, where the risk score and clinicopathological characteristics were combined (Figure 4A). Also, a BC patient was randomly selected for scoring, the results of which are shown in Figure 4A. Calibration curves exhibited good consistency between the nomogram predictions of 1-, 3-, and 5-year OS with the actual observed value (Figure 4B). In order to explore the biological processes of ERS-related lncRNAs, we performed the GO enrichment analysis and GSEA analysis. As shown in Figures 4C, D, in the biological processes (BP), ERS-related lncRNAs were mainly enriched in immune response, B cell activation, and lymphocyte-mediated immunity. In the cellular components (CC), ERS-related lncRNAs were mainly enriched in the immunoglobulin complex and the external side of plasma membrane. In the molecular functions (MF), ERS-related lncRNAs were mainly enriched in antigen binding and immunoglobulin receptor binding. The GSEA analysis (Figure 4E) revealed that the citrate cycle, signaling pathways for glycan and unsaturated fatty acid biosynthesis were mainly enriched in the high-risk groups. For another, in the low-risk groups, immune signaling pathways were more active, such as the T cell receptor signaling pathway and intestinal immune network for IgA production. The JAK STAT signaling pathway was also enriched in the low-risk group.

### Exploration of the correlation between TMB and the ERS-related lncRNA signature

**FIGURE 5:**
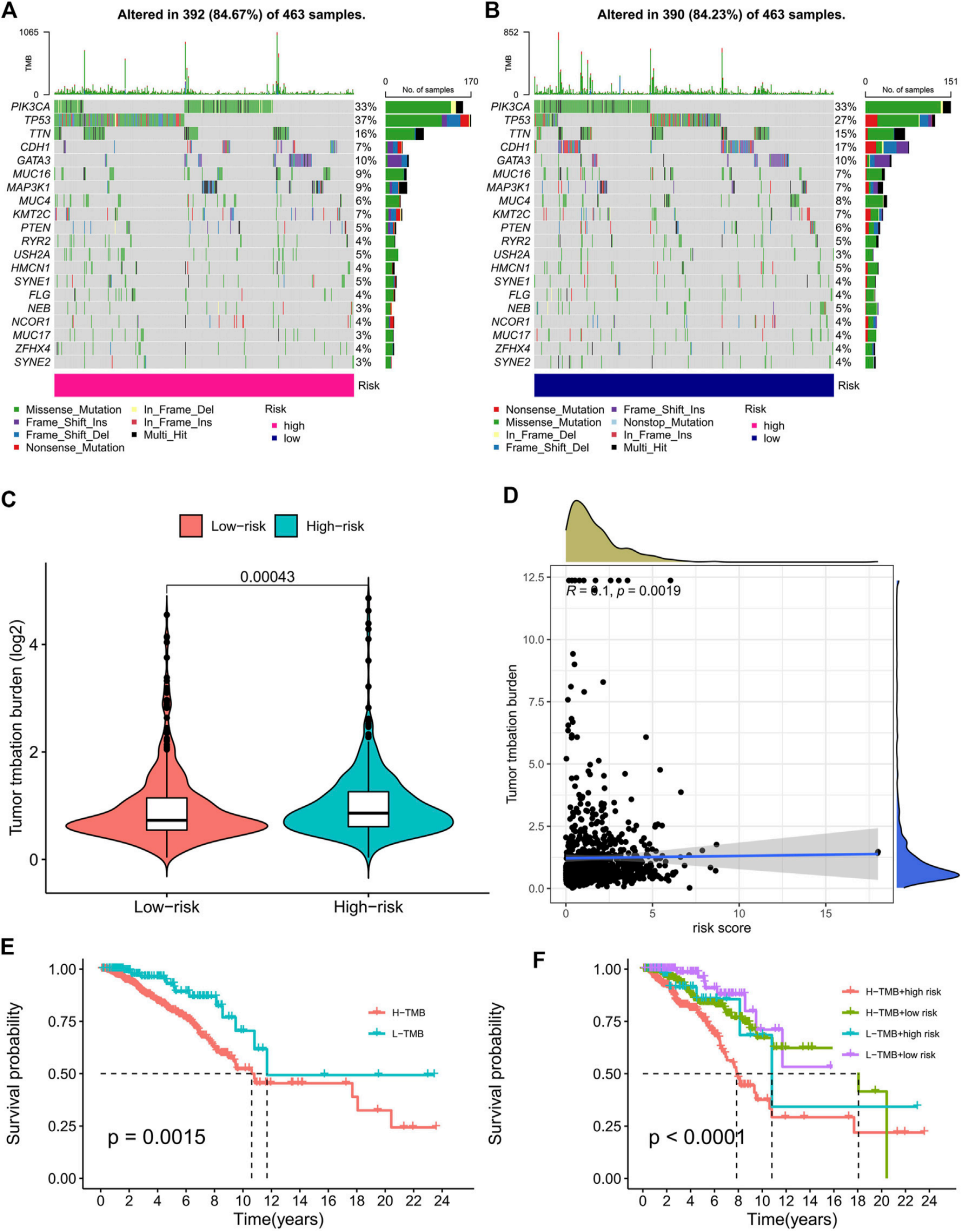
TMB analysis of the prognostic signature. **(A,B)** The waterfall plots of the tumor mutation rate in the high-risk group **(A)** and low-risk group **(B)** based on the prognostic signature. **(C)** The bean plot for the differences in TMB between high- and low-risk groups. **(D)** The correlation curve between TMB and the risk score. **(E)** Kaplan–Meier survival curves of BC patients between the H-TMB and L-TMB groups. **(F)** Kaplan–Meier survival curves of BC patients across H-TMB + high risk, H-TMB + low risk, L-TMB + high risk, and L-TMB + low risk. TMB, tumor mutational burden; H, high; L, low.

Based on the risk score, we used the R package “maftools” to analyze the gene mutation profile of BC patients, including a total of 926 BC samples. The mutation rate in the high-risk group was 84.67% (Figure 5A), whereas it was 84.23% in the low-risk group (Figure 5B). It can be seen from the waterfall charts that the mutated genes in the high- and low-risk groups were mainly PIK3CA, TP53, TTN, CDH1, GATA3, MUC16, MAP3K1, MUC4H, and KMT2C, but the mutation rates of these genes were different except for PIK3CA, GATA3, MUC16, and KMT2C in the high- and low-risk groups. Patients in the high-risk group had higher TMB than those in the low-risk group, as the difference was statistically significant (Figure 5C, *p* = 0.00043). The correlation curve in Figure 5D means that TMB was significantly positively correlated with the risk score (R = 0.1, *p* = 0.0019). It is a TMB survival curve that illustrates low-TMB patients have a better prognosis (Figure 5E, *p* = 0.0015). Compared with the other groups, BC patients with a combination of low risk and low TMB showed the best prognosis (Figure 5F).

### Analyses of the tumor-immune signature

**FIGURE 6:**
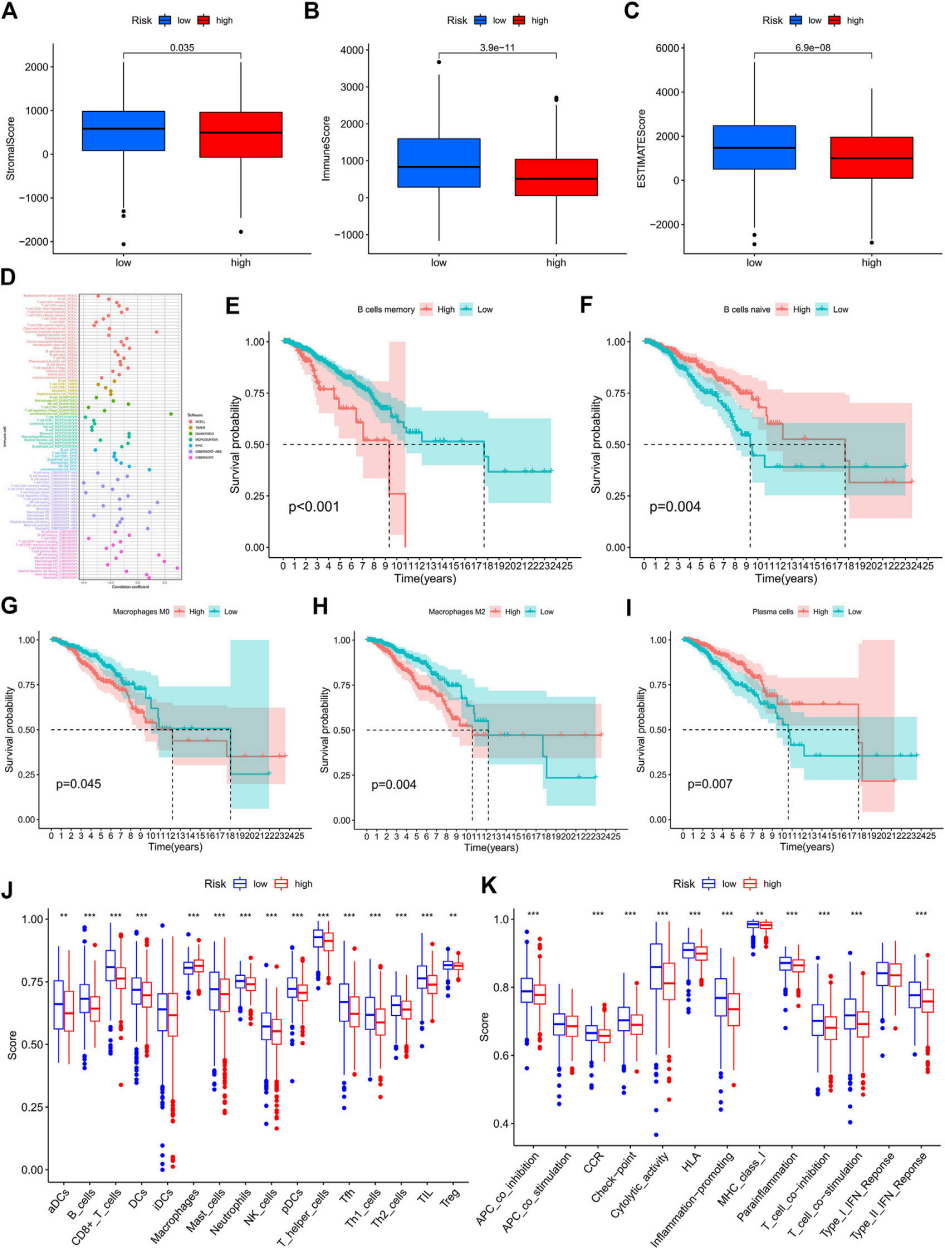
Exploration of the tumor immune status. **(A–C)** The boxplots for StromalScore **(A)**, ImmuneScore **(B)**, and ESTIMATEScore **(C)** in the high- and low-risk groups. **(D)** Estimation of immune-infiltrating cells in BC by using the Spearman correlation analysis. **(E–I)** Kaplan–Meier survival curves of screened B memory cells **(E)**, B naive cells **(F)**, Macrophages M0 **(G)**, Macrophages M2 **(H)** , and plasma cells **(I)** in BC patients. **(J–K)** The score of the infiltrating immune cells **(J)** and immune-related functions **(K)** in the high- and low-risk groups. BC, breast cancer; *, *p* < 0.05; **, *p* < 0.01; ***, *p* < 0.001.

From the scores of the tumor microenvironment in Figures 6A–C, we know that the StromalScore (*p* = 0.035), ImmuneScore (*p* = 3.9e-11), and ESTIMATEScore (*p* = 6.9e-08) of patients in the high-risk group were significantly lower than those in the low-risk group. Using acknowledged methods, we studied immune infiltration fluctuations between the groups. From Figure 6D, we observed the negative correlation coefficients were widespread, implying that patients with a higher classifier index were immunosuppressed. A common lymphoid progenitor in XCELL, uncharacterized cell in QUANTISEQ, uncharacterized cell in EPIC, NK cell resting, macrophage M0 and neutrophil in CIBERSORT-ABS, NK cell resting, macrophage M0, macrophage M2, mast cell resting, and neutrophil in CIBERSORT were negatively correlated with the classifier index, while the other cells in different algorithms showed a positive correlation with the classifier index. In addition, survival analyses showed that as B memory cells (Figure 6E, *p* < 0.001), macrophage M0 (Figure 6G, *p* = 0.045), and macrophage M2 (Figure 6H, *p* = 0.004) infiltration, low-risk patients had significantly higher OS than high-risk patients. Conversely, as B naive cells (Figure 6F, *p* = 0.004) and plasma cells (Figure 6I, *p* = 0.007) infiltrated, high-risk patients had a significantly higher OS than low-risk patients. In previous analyses, we have identified the immunosuppressed status and survival disadvantage in high-risk patients. Along with the ssGSEA enrichment score, we further investigated the relationship between the risk score and different immune cell subsets and functions. For the results, we realized that almost all immune-related functional cells had significantly higher ssGSEA scores in the low-risk patients except in immature dendritic cells (iDCs) (Figure 6J). Similarly, the immune function scores of APC co-inhibition, chemokine receptor (CCR), checkpoint, cytolytic activity, human leukocyte antigen (HLA), inflammation promoting, major histocompatibility complex (MHC) class I, parainflammation, T cell co-inhibition, T cell co-stimulation, and type II IFN responses in the low-risk group were all higher than that in the high-risk group (Figure 6K). Therefore, it prompted the low-risk group to have a higher immune activity.

### Consensus cluster of ERS-related lncRNAs identified three clusters of BC patients

**FIGURE 7:**
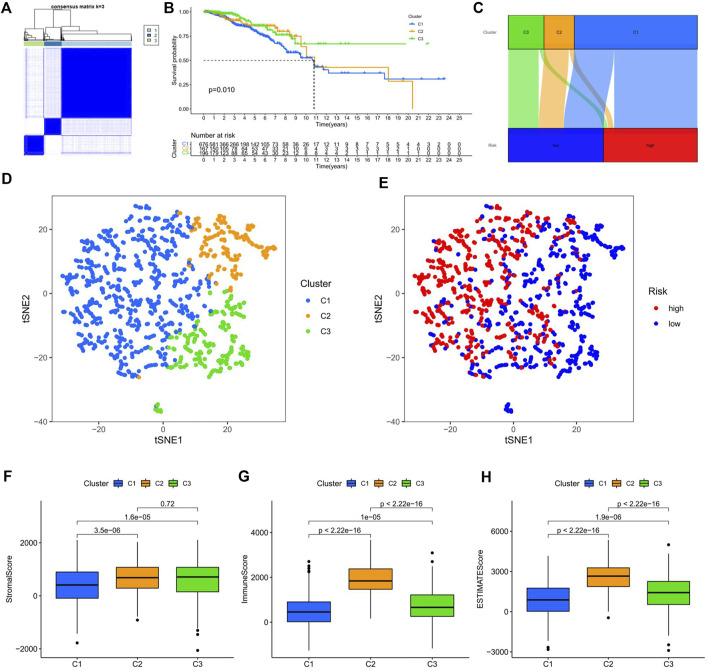
Survival, tSNE, and tumor microenvironment analysis of three distinct subgroups of BC divided by consensus clustering. **(A)** Consensus matrix with optimal k = 3. **(B)** Kaplan–Meier survival curves of BC patients’ OS among the three different subgroups. **(C)** Sankey diagram of the relationship between the three different subgroups and risk score. **(D)** tSNE analysis among the three different subgroups. **(E)** tSNE analysis between the high-risk and low-risk groups. **(F–H)** The boxplots for StromalScore **(F)**, ImmuneScore **(G)**, and ESTIMATEScore **(H)** among the three different subgroups. tSNE, t-Distributed Stochastic Neighbor Embedding; BC, breast cancer; OS, overall survival.

**FIGURE 8:**
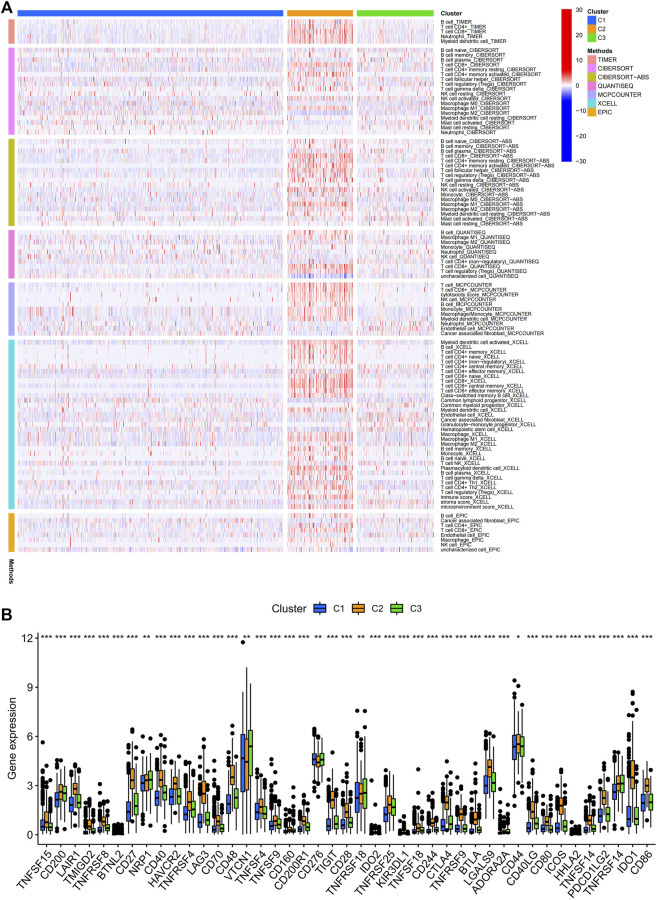
Infiltration of immune cells and the expression of immune checkpoints among the three different subgroups of BC. **(A)** Estimation of immune-infiltrating cells by using the Spearman correlation analysis with multiple algorithms among the three different subgroups. **(B)** Differential expression analysis of 43 immune checkpoint genes among the three different subgroups. BC, breast cancer; *, *p* < 0.05; **, *p* < 0.01; ***, *p* < 0.001.

**FIGURE 9:**
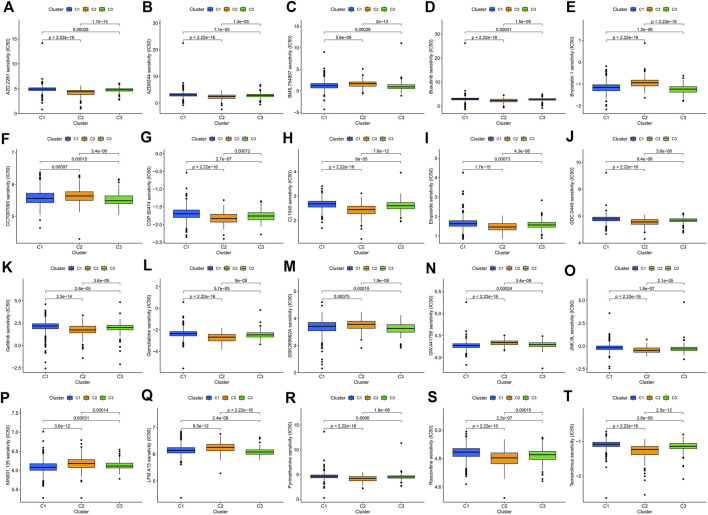
Comparison of potential therapeutic drug susceptibility among the three different subgroups as assessed by IC50. **(A)** AZD.2281. **(B)** AZD6244. **(C)** BMS.754807. **(D)** Bosutinib. **(E)** Bryostatin.1. **(F)** CCT007093. **(G)** CGP.60474. **(H)** CI.1040. **(I)** Etoposide. **(J)** GDC.0449. **(K)** Gefitinib. **(L)** Gemcitabine. **(M)** GSK269962A. **(N)** GW.441756. **(O)** JNK.9L. **(P)** KIN001.135. **(Q)** LFM.A13. **(R)** Pyrimethamine. **(S)** Roscovitine. **(T)** Temsirolimus. Top 20 significantly associations were displayed, as determined by the *p*-value. IC50, the half-maximal inhibitory concentration.

Consensus cluster analysis was performed on the expression level of 9 ERS-related lncRNAs for the sake of probing into the roles of these lncRNAs in BC occurrence and development. k = 3 is an appropriate choice for the most stable aggregation. All tumor samples were divided into 3 subgroups: cluster 1 (*n* = 676), cluster 2 (*n* = 167), and cluster 3 (*n* = 196) (Figure 7A). The OS among patients in the three subgroups had a significant difference (Figure 7B, *p* = 0.010). Cluster 1 was mostly high-risk patients, and clusters 2 and 3 were mostly low-risk patients (Figure 7C). The t-SNE analysis of Figure 7D showed that there were distinct dimensions between different subgroups. Similarly, there were distinct dimensions between the high- and low-risk groups (Figure 7E). As an important role in prognosis, the tumor microenvironment is also worthy of attention. It can be seen that in the StromalScore, there were significant differences between cluster 1 and cluster 2 (*p* = 3.5e-06) and between cluster 1 and cluster 3 (*p* = 1.5e-06), but there was no significant difference between cluster 2 and cluster 3 (*p* = 0.72) (Figure 7F). In the ImmuneScore and ESTIMATEScore, there were significant differences between any two subgroups (Figures 7G,H). The immune response heatmap reflected differences in immune cell infiltration among the three subgroups, showing that cluster 2 had the most immune cell infiltration (Figure 8A). Considering the importance of immunotherapy, we continued to explore the differences in the immune checkpoint molecule expression among the three different subgroups. We can know that the expression of 43 immune checkpoint molecules had significant differences among the three subgroups from Figure 8B. Among them, CD276 has the highest expression in cluster 1, while TNFSF15, CD200, LAIR1, TMIGD2, TNFRSF8, BTNL2, CD27, CD40, HAVCR2, TNFRSF4, LAG3, CD70, CD48, TNFSF4, TNFSF9, CD160, CD200R1, TIGIT, CD28, IDO2, TNFRSF25, KIR3DL1, TNFSF18, CD244, CTLA4, TNFRSF9, BTLA, LGALS9, CD44, CD40LG, CD80, ICOS, TNFSF14, PDCD1LG2, TNFRSF14, IDO1, and CD86 had the highest expression in cluster 2. Also, cluster 3 had the highest expression for NRP1, VTCN1, TNFRSF18, ADORA2A, and HHLA2. Screening of sensitive drugs can lay a good foundation for clinical treatment. By means of a comparative assessment of drug sensitivity by IC50, Figures 9A–T showed 20 drugs whose sensitivities were significantly different between any two subgroups. Notably, BC patients in cluster 1 were more sensitive to GW.441756 (TrkA inhibitor) and KIN001.135 (Lck inhibitor), while those in cluster 3 were more sensitive to BMS.754807(IGF-1R inhibitor), Bryostatin.1, CCT007093(PPM1D inhibitor), GSK269962A (Rho-associated protein kinase inhibitor), and LFM.A13(BTK inhibitor). More drugs have high sensitivities for BC patients in cluster 2, including AZD.2281(Olaparib), AZD6244(Selumetinib), Bosutinib, CGP.60474(PKC inhibitor), CI.1040 (MEK inhibitor), Etoposide, GDC.0449(Vismodegib), Gefitinib, Gemcitabine, JNK.9L (JNK inhibitor), Pyrimethamine, Roscovitine, and Temsirolimus. Thus, it can be seen that BC patients in cluster 2 have more sensitive clinical drugs worthy of selection and development.

### Clinical drug sensitivity analysis and immunotherapy efficacy evaluation of the ERS-related lncRNA prognostic signature

**FIGURE 10:**
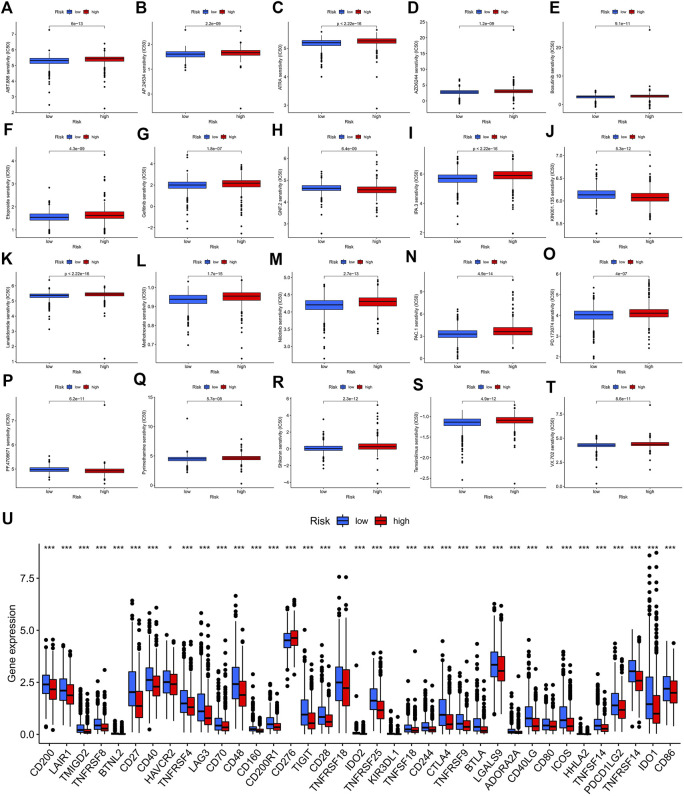
Potential drug sensitivity analysis by IC50 and the immune checkpoint gene expression analysis between the high- and low-risk groups. **(A–T)** The boxplots for the drug sensitivity analysis of **(A)** ABT.888. **(B)** AP.24534. **(C)** ATRA. **(D)** AZD6244. **(E)** Bosutinib. **(F)** Etoposide. **(G)** Gefitinib. **(H)** GNF.2. **(I)** IPA.3. **(J)** KIN001.135. **(K)** Lenalidomide. **(L)** Methotrexate. **(M)** Nilotinib. **(N)** PAC.1. **(O)** PD.173074. **(P)** PF.4708671. **(Q)** Pyrimethamine. **(R)** Shikonin. **(S)** Temsirolimus. **(T)** VX.702. Top 20 significantly associations were displayed, as determined by the *p*-value. **(U)** Differential expression analysis of the immune checkpoint genes between the high-risk and low-risk groups. IC50, the half-maximal inhibitory concentration; *, *p* < 0.05; **, *p* < 0.01; ***, *p* < 0.001.

With broad study prospects, the drug treatment of BC has attracted much attention. We calculated the IC50 of drugs on BC to analyze the relationship between risk scores and drug resistance. We noted that IC50 of GNF.2 (Bcr-Abl inhibitor), KIN001.135 (Lck inhibitor), and PF.4708671 (S6 kinase inhibitor) in high-risk patients were higher, while all other drugs had higher IC50 in the low-risk patients (Figures 10A–T). We further identified the expression of immune checkpoint genes between high- and low-risk groups in consideration of the clinical application and benefits of ICIs. As a result, only CD276 was more highly expressed in the high-risk group compared to the low-risk group, while the expression levels of all other immune checkpoint genes were significantly higher in the low-risk group than those in the high-risk group (Figure 10U).

## Discussion

Viewing the bioinformatics analysis as a foundation, identifying biomarkers through database mining can predict the prognosis of BC (Ping et al., 2021). LncRNAs are abnormally expressed in a wide range of cancers (Huarte, 2015), and it has been demonstrated that lncRNAs are involved in tumor-related cellular pathways, resulting in good predictive power in terms of diagnosis and prognosis (Chi et al., 2019). To the best of our knowledge, our study is the first comprehensive elaboration of the relationship between ERS-related lncRNAs and BC, starting from prognostic identification, biological pathways, TMB, tumor immune microenvironment, cluster, and clinical treatment. It can provide an important reference for future in-depth research on prognosis prediction and clinical individualized treatment of BC patients.

ERS is a condition triggered by mis-folded or unfolded proteins in the endoplasmic reticulum and is directed by activating transcription factor 6 (ATF6), inositol-requiring enzyme 1α (IRE1α), and protein kinase RNA-like endoplasmic reticulum kinase (PERK) mediated by signaling pathways (Akman et al., 2021). In this study, we screened out the 9 best ERS-related lncRNAs (LINC02446, JARID2-AS1, AC025259.3, AC105046.1, TFAP2A-AS1, AC022898.2, AC005757.1, TBL1XR1-AS1, and AC137932.2) as a prognostic model. Training, validation, and complete sets of survival curves, risk score plots, survival status plots, and heatmaps were used to assess the prognostic value of signatures. Clearly, the prognostic model has a good predictive value. It can be seen that the OS of patients in the high-risk group is lower than that in the low-risk group, reflecting that ERS as a cancer-promoting factor affects the prognosis of BC, which is consistent with the results of previous studies (Jiang et al., 2022). A series of data also suggest that ER stress can promote tumor progression pass through multiple mechanisms including cancer cell survival and metastasis, therapy resistance, and angiogenesis (Lee, 2007; Urra et al., 2016). Univariate and multivariate analyses suggested that the risk score could be used as an independent prognostic factor for BC patients (*P*< 0.001). The AUC value ​​of the prognostic signature was 0.742 and the AUC values of 1-, 3-, and 5-year predicted survival rates were 0.742, 0.703, and 0.645, respectively, which not only showed the better predictive performance compared with other clinicopathological characteristics (gender, stage, T stage, N stage, and M stage), but also reflected the reliability and precision of the prognostic signature. BC patients in the low-risk group with different clinicopathological characteristics had significantly better OS, except for the M1 stage. More importantly, the calibration curves showed an excellent concordance between the 1-, 3-, and 5-year survival rates predicted by the prognostic signature and the actual OS rates. Taken together, the model we constructed can be considered as a good prognostic signature whose mechanism of action in BC deserves further exploration and validation.

By the GO enrichment analysis, we noticed that immune response, B cell activation, and lymphocyte-mediated immunity play key roles in the biological pathways of ERS. From the GSEA enrichment analysis, we found that high-risk patients have abundant metabolic pathways, such as citrate cycle, signaling pathways for glycan and unsaturated fatty acid biosynthesis. Needless to say, tumor progression is closely related to the metabolism. IRE1-XBP1(X-box binding protein 1) signaling is involved in the reprogramming of cancer metabolism during endoplasmic reticulum stress (Chen and Cubillos-Ruiz, 2021). XBP1 directly induces fatty acid synthase and citrate lyase to regulate lipid metabolism (Lee et al., 2008; Xie et al., 2018). It can be speculated that for ERS, BC in the high-risk group is more likely to promote tumor progression by regulating tumor metabolism, especially fatty acid metabolism, which is worthy of further exploration as it is expected to be a new target for treatment. In contrast, immune signaling pathways are more active in low-risk patients. It can be seen that immune surveillance inhibits the cancer-promoting effect of ERS, which provides a theoretical basis for the study of immunotherapy.

Studies have shown that TMB is a reliable biomarker for predicting the treatment outcome in cancer patients treated with ICIs (Cao et al., 2019). Our study showed that the mutation rate of gene TP53 was highest in high-risk patients, while the mutation rate of gene PIK3CA was highest in low-risk patients. Mutations in the TP53 gene are associated with a poor treatment effect and prognosis in BC (Hu et al., 2018; Takahashi et al., 2021). TMB was significantly associated with the risk score. High-risk patients had higher TMB and a worse OS. For the immune cell and stromal cell fractions, we can predict that both of them play an important role in the effects of TIME. Risk scores were inversely associated with most tumor-infiltrating immune cells. Infiltration of B memory cells, macrophage M0, and macrophage M2 resulted in a higher OS in low-risk patients, whereas B naïve cells and plasma cells resulted in a higher OS in high-risk patients. B memory cells function as antigen-presenting cells in both naive and memory T cell responses, inducing anti-tumor immune responses to kill tumor cells (Helmink et al., 2020). By using ssGSEA, we probed the immune status of different groups, revealing that the low-risk group had more immune cell infiltrations and a stronger anti-tumor immunity. Studies have shown that the intrinsic ERS responses of cancer cells can influence the malignant progression by altering the function of immune cells co-existing in the tumor microenvironment (Chen and Cubillos-Ruiz, 2021). How immune functions such as T cell co-inhibition, T cell co-stimulation, type II IFN responses, and so on affect the survival of tumor cells deserve further exploration. As a consequence, we speculate that one of the reasons for the poor prognosis of high-risk BC patients may be due to low immune cell infiltration and low anti-tumor immune function.

Next, we divided the BC patients into 3 subgroups to explore the relationship among tumor subtypes. Cluster 2, mainly consisting of low-risk patients, had the highest immune score and the most immune cell infiltrations, which reflects the most active immune function in cluster 2. According to the study of Duan et al., we know that CD8^+^ T cells can kill cancer cells, break immune tolerance, and promote immunotherapy pass through the PD-1/PD-L1 immunosuppressive axis (Duan et al., 2020). Cluster 2 had the highest degree of CD8^+^ T cell infiltration among the three subgroups, and had high expression of the most immune checkpoint molecules. It can be speculated that BC patients in cluster 2 may have a better clinical immune efficacy. It is important to study the relationship between subgroups and sensitivity to molecularly targeted and chemotherapeutic agents. We found that BC patients in cluster 2 were most sensitive to some of the drugs already in clinical application such as olaparib, etoposide, and gemcitabine. The OlympiAD study showed that Olaparib significantly prolonged PFS compared with chemotherapy in patients with BRCA-mutated HER-2-negative advanced BC (Robson et al., 2017; Robson et al., 2019). Gemcitabine can be used as a single agent in the treatment of advanced BC (Seidman, 2001). For those drugs that have not been applied to BC in clinics, our research provides a certain theoretical basis for their development. The aforementioned results suggest that, for one thing, tumor subtype may be a potential predictor to guide targeted therapy and chemotherapy in BC patients; for another, ERS may have high research value in the late progression of BC.

Oncology treatment is an important area of concern. Through the IC50 screening analysis of potential drugs, we realized that high-risk patients may be sensitive to the Bcr-Abl inhibitor, Lck inhibitor, and S6 kinase inhibitor, but may be resistant to ABT.888 (Veliparib), AP.24534(Ponatinib), ATRA (All-Trans Retinoic Acid), AZD6244 (Selumetinib), Bosutinib, Etoposide, Gefitinib, IPA.3 (Pak1 inhibitor), Lenalidomide, Methotrexate, Nilotinib, PAC.1 (procaspase-3 activator), PD.173074 (FGFR1 inhibitors), Pyrimethamine, Shikonin, Temsirolimus and VX.702 (p38α MAPK inhibitor). Hypoxia and UPR act synergistically in inducing chemoresistance. ATF6, IRE1α, and PERK are jointly involved in chemoresistance (Akman et al., 2021), prompting that it is of interest to explore potential links between drugs and them. In addition, some immunotherapy drugs have been applied in triple-negative BC (Cortes et al., 2020), but not all BC patients can benefit from them (Adams et al., 2019; TCGA, 2022). And, more scholars have proposed that the combination of TMB and other biomarkers can better predict the efficacy of tumor immunotherapy. Therefore, based on the ERS-related lncRNA prognostic signature, we found that low-risk patients are sensitive to the vast majority of immunotherapy drugs and may have a better efficacy. We speculate that low-risk patients are more favorable for inducing anti-tumor immune responses and are more likely to benefit from immunotherapy. In summary, on one hand, we think out the ERS-related lncRNA prognostic signature can serve as a new indicator for evaluating the applicability of ICIs. On the other hand, combined with drug sensitivity and immune efficacy analyses, we predict that low-risk patients will benefit more from the combination of chemotherapy, targeted therapy, and immunotherapy, providing a basis for the individualized treatment of BC patients. And meanwhile, there are also more drugs worthy of selection and development for low-risk patients. Beyond all doubt, our study has the following limitations. First, the prognostic signature is based on the TGCA public database, lacking validation of samples from other databases. Second, our study confirms that the prognostic signature has a good predictive value, but *in vitro* basic experiments are still needed to confirm the mechanism of ERS in BC. Third, the relationship between prognostic characteristics and the effects of targeted therapy, chemotherapy, and immunotherapy in BC patients need to be urgently studied through a large number of clinical trials.

This study constructed an ERS-related lncRNA prognostic signature, and described its correlation with TMB, immunity, and clinical treatments (targeted therapy, chemotherapy, and immunotherapy), which can provide reference for the individualized and precise treatment of BC.

## Data Availability

The original contributions presented in the study are included in the article/Supplementary Material; further inquiries can be directed to the corresponding authors.
