# Supplementary material for: Development and validation of a novel endoplasmic reticulum stress-related lncRNA prognostic signature and candidate drugs in breast cancer
Source: Front Genet. 2022 Aug 25;13:949314. doi: 10.3389/fgene.2022.949314 (PMC9452962; doi:10.3389/fgene.2022.949314)
Supplement: Supplementary file 3 [file Table1.DOCX]

Table 1 | The regression coefficient of 9 ESR-related lncRNAs acquired by the multivariate Cox analysis.

| id | coef |
| --- | --- |
| LINC02446 | -0.666557169115816 |
| JARID2-AS1 | -1.01852389169384 |
| AC025259.3 | -0.494972345670693 |
| AC105046.1 | -1.70783864216911 |
| TFAP2A-AS1 | -0.549326255865056 |
| AC022898.2 | -2.95365678990592 |
| AC005757.1 | -2.66162430189308 |
| TBL1XR1-AS1 | -3.86616790061961 |
| AC137932.2 | 2.26501183795505 |
