# Supplementary material for: Development and validation of a novel endoplasmic reticulum stress-related lncRNA prognostic signature and candidate drugs in breast cancer
Source: Front Genet. 2022 Aug 25;13:949314. doi: 10.3389/fgene.2022.949314 (PMC9452962; doi:10.3389/fgene.2022.949314)
Supplement: Supplementary file 5 [file DataSheet1.docx]

Figure 1 | Identification of prognostic ERS-related lncRNAs for BC patients. (A) Heatmap of 145 differentially expressed lncRNAs. (B) Volcano plot of ERS-related lncRNAs. Red dots indicate up-regulated lncRNAs in tumor tissue while green dots indicate down-regulated lncRNAs. (C, D) Forest plot (C) and heatmap (D) of prognostic ERS-related lncRNAs extracted by univariate Cox regression analysis. (E, F) LASSO variable trajectory plot for 1000 cross validations(E) and LASSO coefficient profile (F). ERS, endoplasmic reticulum stress; BC, breast cancer.

Figure 2 | Prognosis and risk scoring analysis of 9 ERS-related lncRNAs in the different sets for BC patients. (A-C) Kaplan-Meier survival curves of BC patients’ OS in the high- and low-risk groups in the training(A), validation(B) and complete sets(C). (D-F) Risk score distribution in the training(D), validation(E) and complete sets(F) for two groups. (G-I) Scatter plots of BC patients’ survival status distribution in the training(G), validation(H) and complete sets(I). (J-L) Risk heatmaps of the 9 ERS-related lncRNAs expression in the training(J), validation(K) and complete sets(L). Red represents high expression and green represents low expression. ERS, endoplasmic reticulum stress; BC, breast cancer; OS, overall survival.

Figure 3 | Validation of the ERS-Related lncRNAs prognostic signature and its relationship with clinicopathological characteristics. (A, B) Univariate (A) and multivariate (B) independent Cox regression analyses of risk score and clinicopathological characteristics. (C) ROC curves and AUCs of risk score and clinicopathological characteristics. (D) ROC curves and AUCs for 1-, 3-, and 5-year survival of the complete set. (E-L) Kaplan-Meier survival curves of BC patients’ OS on clinicopathological characteristics between two groups in the complete set. ERS, endoplasmic reticulum stress; AUC, area under the curve; ROC, receiver operating characteristic; OS, overall survival; T, tumor; N, lymph node; M, metastasis.

Figure 4 | Construction of the nomogram and calibration curves, enrichment analysis of the prognostic signature. (A) nomogram prediction model of combined risk score and clinicopathological characteristics for 1-, 3-, and 5-year OS in BC patients. (B) Calibration curves for the relationship between predicted survival and observed OS rate at 1, 3, and 5 years. (C-D) The GO function enrichment analyses. (E) The GSEA pathways enrichment analyses. OS, overall survival; BC, breast cancer.

Figure 5 | TMB analysis of prognostic signature. (A, B) The waterfall plots of the tumor mutation rate in high-risk group (A) and low-risk group (B) based on the prognostic signature. (C) The bean plot for differences in TMB between high- and low-risk groups. (D) The correlation curve between TMB and risk score. (E) Kaplan-Meier survival curves of BC patients between H-TMB and L-TMB groups. (F) Kaplan-Meier survival curves of BC patients across H-TMB+ high risk, H-TMB+ low risk, L-TMB+ high risk, and L-TMB+ low risk. TMB, tumor mutational burden; H, high; L, low.

Figure 6 | Exploration of the tumor immune status. (A-C) The boxplots for StromalScore (A), ImmuneScore (B) and ESTIMATEScore (C) in high- and low-risk groups. (D) Estimation of immune-infiltrating cells in BC by Spearman correlation analysis. (E-I) Kaplan-Meier survival curves of screened B memory cells(E), B naive cells(F), Macrophages M0 (G), Macrophages M2 (H)and plasma cells (I) in BC patients. (J-K) The score of infiltrating immune cells(J) and immune-related functions(K) in the high- and low-risk groups. BC, breast cancer; *, *p* < 0.05; **, *p* < 0.01; ***, *p* < 0.001.

Figure 7 | Survival, tSNE and tumor microenvironment analysis of three distinct subgroups of BC divided by consensus clustering. (A) Consensus matrix with optimal k=3. (B) Kaplan-Meier survival curves of BC patients’ OS among three different subgroups. (C) Sankey diagram of the relationship between three different subgroups and risk. (D) tSNE analysis among three different subgroups. (E) tSNE analysis between high-risk and low-risk groups. (F-H) The boxplots for StromalScore (F), ImmuneScore (G) and ESTIMATEScore (H) among three different subgroups. tSNE, t-Distributed Stochastic Neighbor Embedding; BC, breast cancer; OS, overall survival.

Figure 8 | Infiltration of immune cells and expression of immune checkpoints among three different subgroups of BC. (A) Estimation of immune-infiltrating cells by Spearman correlation analysis with multiple algorithms among three different subgroups. (B) Differential expression analysis of 43 immune checkpoint genes among three different subgroups. BC, breast cancer; *, *p* < 0.05; **, *p* < 0.01; ***, *p* < 0.001.

Figure 9 | Comparison of potential therapeutic drug susceptibility among three different subgroups as assessed by IC50. (A)AZD.2281. (B)AZD6244. (C)BMS.754807. (D)Bosutinib. (E)Bryostatin.1. (F)CCT007093. (G)CGP.60474. (H)CI.1040. (I)Etoposide. (J)GDC.0449. (K)Gefitinib. (L)Gemcitabine. (M)GSK269962A. (N)GW.441756. (O)JNK.9L. (P)KIN001.135. (Q)LFM.A13. (R)Pyrimethamine. (S)Roscovitine. (T)Temsirolimus. Top 20 significantly associations were displayed, as determined by the *p*-value. IC50, the half-maximal inhibitory concentration.

Figure 10 | Potential drug sensitivity analysis by IC50 and immune checkpoint gene expression analysis between high- and low-risk groups. (A-T) The boxplots for drug sensitivity analysis of (A) ABT.888. (B) AP.24534. (C) ATRA. (D) AZD6244. (E) Bosutinib. (F) Etoposide. (G) Gefitinib. (H) GNF.2. (I) IPA.3. (J) KIN001.135. (K) Lenalidomide. (L) Methotrexate. (M) Nilotinib. (N) PAC.1. (O) PD.173074. (P) PF.4708671. (Q) Pyrimethamine. (R) Shikonin. (S) Temsirolimus. (T) VX.702. Top 20 significantly associations were displayed, as determined by the *p*-value. (U) Differential expression analysis of immune checkpoint genes between high-risk and low-risk groups. IC50, the half-maximal inhibitory concentration; *, *p* < 0.05; **, *p* < 0.01; ***, *p* < 0.001.
